# *Francisella tularensis-*specific antibody levels in sera from Swedish patients with suspected tularemia during a 13-year period

**DOI:** 10.3389/fcimb.2024.1381776

**Published:** 2024-04-02

**Authors:** Helena Lindgren, Xijia Liu, Anders Sjöstedt

**Affiliations:** ^1^ Department of Clinical Microbiology, Umeå University, Umeå, Sweden; ^2^ Umeå School of Business, Economics and Statistics, Umeå University, Umeå, Sweden

**Keywords:** tularemia, serological response, annual distribution, monthly distribution, age-related titers

## Abstract

**Introduction:**

For a majority of tularemia patients, serology is the basis for the diagnosis. The aim of this study was to perform an analysis of the samples analyzed at a Swedish reference laboratory for the presence of Francisella tularensis-specific antibody levels in sera from individuals with suspected tularemia. Annual and monthly variations of the total number of samples and proportions of positive samples were analyzed, as well as the influence of age and gender.

**Methods:**

We performed a retrospective analysis of the presence of F. tularensis-specific antibodies in serological samples from patients with suspected tularemia analyzed during the period 2010 - 2022 at the University Hospital of Umeå in Sweden, a national reference laboratory, by use of various statistical methods. In total, some 15,100 serum samples had been analyzed for the presence of IgG and IgM antibodies by ELISA during the 13-year period.

**Results:**

Overall, there were higher number of samples with IgG positive or borderline titers, 2,522 and 921, respectively, than with IgM positive or borderline titers, 1,802 and 409, respectively. Repeated samples were obtained from some 1,930 individuals and approximately a third of the cases, which were initially seronegative, had seroconverted when resampled. Peak number of monthly samples were recorded in August and September, > 3,000. Annual numbers varied greatly and peak numbers were observed in 2015 and 2019, 1,832 and 2,250, respectively, whereas some other years the numbers were 700 – 800. There was also much variation in the annual and monthly percentages of positive samples and they varied between less than 10% to greater than 20%. The highest percentages of positive samples were recorded in September and October. IgG and IgM titers declined with age and these differences were highly significant for IgG titers, with decreasing average titers for each 20-year interval.

**Discussion:**

Collectively, the data demonstrate the marked annual and seasonal variations in tularemia sampling occurring in Sweden. Also, the proportion of positive samples increased during months and years with peak number of samples. Another notable finding was that average antibody titers decreased with increased age.

## Introduction

*Francisella tularensis* is a Gram-negative intracellular bacterium. The bacterium is highly contagious and causes the zoonotic disease tularemia in humans as well as in many other mammalian species (Sjöstedt, 2007; Petersen et al., 2017). Tularemia has been described in many countries of the Northern hemisphere and in a few of the Southern hemisphere (Petersen et al., 2017). In most countries, it is a rare disease, but it is more frequently occurring in some European countries, in particular Sweden, Finland, Turkey, Hungary, and Czech Republic (Hestvik et al., 2015). Generally, and including the aforementioned countries, the disease shows very marked annual and seasonal variations, and most outbreaks are local with random occurrence, but predominantly during late summer and autumn (Tärnvik et al., 2004; Maurin and Gyuranecz, 2016).

The severity of tularemia is dependent on the infecting subspecies. Globally, subspecies *holarctica* is the most common and it is spread over the whole Northern hemisphere (Sjöstedt, 2007; Petersen et al., 2017). This causes a potentially severe disease, particularly the respiratory form, but even without treatment, fatality is very rare, estimated to be 1% (Tärnvik and Berglund, 2003). In contrast, subspecies *tularensis*, is distinctly more virulent. This subspecies is only found in North America with an estimated case fatality estimated to be 10% if untreated (Staples et al., 2006). Regardless of subspecies, *F. tularensis* is highly infectious and the infecting dose is estimated to be 10 bacteria or fewer (Petersen et al., 2017). Due to its high virulence, low infectious dose, and aerosol transmission, it has been classified as a potential bioterrorism agent.

General symptoms of tularemia are flu-like, such as fever, myalgia, and headache, and these normally develop with an incubation period of 3 - 5 days, but there are also additional symptoms dependent on the route of entry (Tärnvik and Berglund, 2003; Maurin and Gyuranecz, 2016). The predominant form of disease, ulceroglandular tularemia, presents with an ulceration at the inoculation site, followed by prominent lymphadenopathy (Tärnvik and Berglund, 2003). This form is a result of vector-borne infection. In most parts of the world, ticks are the predominant vector, however, in Scandinavia, mosquitos constitute the most common vector (Maurin and Gyuranecz, 2016). The second common form is respiratory tularemia, which results from the inhalation of contaminated dust, and it presents with fever, cough, enlarged mediastinal lymph nodes, and pneumonia (Tärnvik and Berglund, 2003; Petersen et al., 2017). More severe variants of the latter form may result in respiratory distress and, sometimes, fatal outcome. Less common forms include oculoglandular, oropharyngeal tularemia, and meningitis. The former two occur after conjunctival or pharyngeal inoculation, respectively. Sometimes, also typhoidal tularemia is described. It designates a systemic disease with high fever, but no evident portal of entry (Tärnvik and Berglund, 2003). Likely, this form is often related to respiratory disease with systemic spread.

Since initial prominent symptoms during ulceroglandular and respiratory tularemia are non-specific, early diagnosis is challenging. PCR-based methods for detection of *F. tularensis* in exudates from ulcers are useful, however, the infected ulcer is not always identified, so for a majority of patients, diagnosis is based on serology (Chaignat et al., 2014; Maurin and Gyuranecz, 2016; Maurin, 2020). In view of the slow kinetics of the serological response, confirmation usually occurs at earliest during the second week (Yanes et al., 2018). Noteworthy, the humoral immune response after eradication of tularemia, is unusually long-lasting, *e.g*., elevated IgM antibodies have been reported to be present 11 years after infection and low, but significant IgG antibody titers detectable in 50% of individuals up to 25 years after infection (Koskela and Salminen, 1985; Ericsson et al., 1994). In a recent study, it was observed that a majority of individuals exhibited significantly elevated IgG and IgM titers 12 months after infection (Lindgren et al., 2022). Thus, low antibody titers are of limited diagnostic value since they are not indicative of ongoing tularemia.

The present study was a retrospective analysis of the presence of *F. tularensis*-specific antibodies in samples from patients with suspected tularemia analyzed during the period 2010 - 2022 at the University Hospital of Umeå in Sweden. The analysis demonstrates marked annual and seasonal variations in tularemia sampling occurring in Sweden, reflecting the pronounced variation in actual number of tularemia cases between and during years, and also that the proportion of positive samples increased during months and years with peak number of samples. The data also demonstrate that average antibody titers decreased with increased age.

## Materials and methods

### Data collection

The study was a retrospective analysis and involved all serum samples analyzed for the presence of *Francisella*-specific antibodies at the University Hospital of Umeå during 2010 - 2022, the period for which there are electronic records available. Serum samples were received from many parts of Sweden since the laboratory, together with the Public Health Agency of Sweden, serves as a national reference laboratory for tularemia. The total number of samples was approximately 15,100 and the data was retrieved from the laboratory system CGM Analytix (CompuGroup Medical Sweden, Solna, Sweden). The results were classified as positive, negative, or borderline depending on the specific antibody titers. In addition, information was available regarding the absolute levels of each antibody titer, the patient’s age at the time of sampling, the gender, and the sample collection date. Ethical approval for the study was received from the Swedish Ethical Review Authority, 2023-05165.

### Reagents

Goat anti-human IgM- and IgG-alkaline phosphatase antibodies and NUNC MaxiSorp plates were purchased from Sigma Aldrich, Darmstadt, Germany. Highly purified *F. tularensis* LPS was a generous gift from Dr. Wayne Conlan, NRC-CNRC, Ottawa, Canada. The LPS was purified as described (Vinogradov et al., 2002).

### Measurement of *F. tularensis*-specific IgM and IgG antibody titers

An ELISA was used to determine IgM and IgG antibody titers. The wells of Nunc MaxiSorp plates (Sigma Aldrich) were coated with 100 µl of *F. tularensis* LPS (12 µg/ml) diluted in 0.05 M of carbonate-bicarbonate, pH 9.5 and incubated overnight at 26°C. The antigen was removed and wells filled with PBS + BSA 0.5% and stored at -80°C until use. Uncoated wells were included to control for non-specific binding. Before addition of serum samples, the plates were washed four times with PBS + 0.05% BSA. The patient sera were diluted 1,000-fold in incubation buffer (PBS + 0.05% of Tween-20). One hundred µl of the diluted serum was added to coated and uncoated wells. The plates were incubated at 26°C for 3 h and thereafter washed. The anti-IgG- and anti-IgM-conjugated alkaline phosphatase antibodies were diluted 5,000-fold in incubation buffer and 100 µl added to each well. After overnight incubation at 26°C, plates were washed three times before 100 µl of alkaline phosphatase substrate was added to each well. The reaction was stopped by adding 50 µl of 3.0 M of NaOH per well when wells incubated with the calibrator serum had reached an OD of 1.0 A_405_. The relative titer of each serum sample was calculated by subtracting the value of the non-coated wells and thereafter multiplying the value with the correction factor and the dilution factor. The correction factor was calculated by dividing the nominal value of the calibrator serum, 1.0, with the obtained value of wells with the calibrator serum. Based on statistical analyses of data from an extensive collection of sera from donors with no known exposure to *F. tularensis*, 100 - 200 was determined to be a threshold value and a value of > 200 as positive for both IgG and IgM.

### Titration of calibrator serum

A calibrator serum was included in each ELISA plate to normalize data. The serum had been collected from a person vaccinated with *F. tularensis* LVS and tested for antibody titers using two-fold serial dilutions, 100-12,800, and an appropriate dilution was selected as a calibrator serum.

### Statistical analysis

SPSS version 29 was used. Two-sided Student`s *t*-test or One-Way ANOVA with Bonferroni correction were used to compare differences among groups.

## Results

### Analysis of *F. tularensis*-specific IgG and IgM antibody levels

Serum samples collected from patients in Sweden with suspected tularemia during the period 2010 to 2022 were analyzed for the presence of *F. tularensis*-specific IgG and IgM antibodies. Details regarding the sera classified as positive or borderline are presented in **Table 1**. In total, some 15,100 samples were analyzed during the 13-year period. A large majority of the sera demonstrated no significant IgG or IgM titers; 77.2% showed no IgG reactivity and 85.4% showed no IgM reactivity (**Table 2**). Among the remaining samples, 2,522 (16.7%) were IgG positive and 921 (6.1%) IgG borderline, whereas 1,802 (11.9%) were IgM positive and 409 (2.7%) IgM borderline.

**Table 1:** Overview of the positive or borderline serological results.

Serological group	Median	Max	SEM	Skewness
IgG				
Borderline	130	200	0.9	0.4
Positive	600	3,215	7.3	1.1
IgM				
Borderline	140	200	1.4	0.1
Positive	595	1,880	7.6	0.6

**Table 2:** Total number of sera in each serological category.

	IgG (n)	IgM (n)
Negative	11,654 (77.2%)	12,891 (85.4%)
Positive	2,522 (16.7%)	1,802 (11.9%)
Borderline	921 (6.1%)	409 (2.7%)
Total	15,097	15,102

A further analysis of the data identified some 1,930 instances of repeated sampling from the same patients (**Tables 3A, B**). In 74.2% of the cases, the original samples were IgG negative and among these, approximately a third showed a borderline or positive titer upon resampling (**Table 3A**). When IgG titers in the original sample were at borderline levels, approximately half of the repeated samples showed borderline and half positive IgG titers. An absolute majority of cases originally IgG positive showed the same result upon resampling (**Table 3A**). The time to resampling was analyzed and did not differ between samples from individuals that seroconverted and those that did not seroconvert, 30.8 *vs*. 28.6 days, respectively (*P* < 0.22). The average age of the individuals in the two groups did not differ, 54.8 *vs*. 55.2 years, respectively (*P* < 0.70).

**Table 3:** A. Results of repeated sampling for IgG *Francisella*-reactivity.

Original IgG result		Repeated IgG result		
		Negative^1^	Borderline^2^	Positive^3^
Negative	1,436^4^ (74.2%)^5^	944 (48.84%)	93 (4.8%)	399 (20.6%)
Borderline	190 (9.8%)	25 (1.3%)	78 (4.0%)	87 (4.5%)
Positive	310 (16%)	7 (0.4%)	19 (1.0%)	284 (14.7%)
Total	1,936 (100%)	976 (50.4%)	190 (9.8%)	770 (39.8%)

^1^Titer below 100.

^2^Titer between 100 and 200.

^3^Titer above 200.

^4^number of observations in the specific serological category.

^5^Percentage observations in the specific serological category.

**Table 3:** B. Results of repeated sampling for IgM *Francisella*-reactivity.

Original IgM result		Repeated IgM result		
		Negative^1^	Borderline^2^	Positive^3^
Negative	1,689^4^ (87.9%)^5^	1,163 (60.5%)	61 (3.2%)	465 (24.2)
Borderline	62 (3.2%)	10 (0.5%)	13 (0.7%)	39 (2.0%)
Positive	170 (8.8%)	11 (0.6%)	12 (0.6%)	147 (7.7%)
Total	1,921 (100%)	486 (25.3%)	86 (4.5%)	651 (33.9%)

^1^Titer below 100.

^2^Titer between 100 and 200.

^3^Titer above 200.

^4^number of observations in the specific serological category.

^5^Percentage observations in the specific serological category.

In 87.9% of the cases, the original samples were IgM negative, and of these, approximately a third showed a borderline or positive titer upon resampling (**Table 3B**). The number of original samples with titers of IgM at border line was very low and most were positive upon resampling. Almost all of the cases that were originally IgM positive, showed the same repeated result (**Table 3B**). The time to resampling was analyzed and did not differ between samples from individuals that seroconverted and those that did not seroconvert, 31.2 *vs*. 28.4 days, respectively (*P* < 0.09). The average age of the individuals in the two groups did not differ, 55.9 *vs*. 55.4 years, respectively (*P* < 0.62).

Thus, approximately a third of the cases, which were initially seronegative, had seroconverted when resampled.

### Monthly and annual distribution of *F. tularensis*-specific IgG and IgM antibody titers

**Figure 1:**
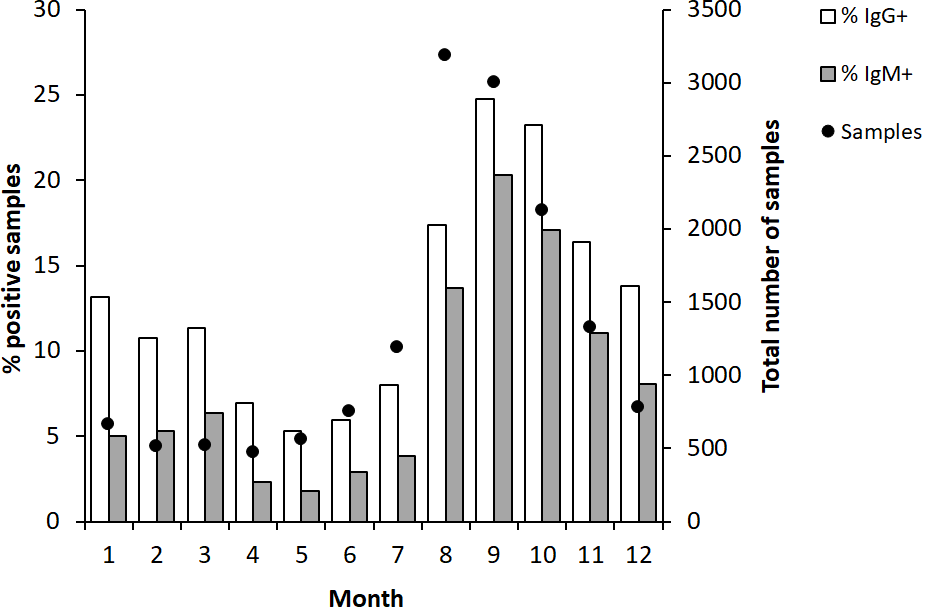
Monthly proportion of IgG and IgM positive serum samples (bars) and numbers of serum samples analyzed each month (dot). The serum samples were collected from patients in Sweden with suspected tularemia during the period 2010 to 2022.

During the 12-year period, the accumulated monthly samples peaked in August and September, > 3,000, whereas between 1,000 to 2,000 samples had been analyzed in July, October, and November. During the remainder of the year, there were less than 800 monthly samples (**Figure 1**). The percentage of IgG positive samples peaked in September and October, > 20%, whereas there were 10 -15% positive samples from November to March and in August. From April until July, there were less than 10% IgG-positive samples (**Figure 1**).

A similar trend as described for IgG was observed for IgM-positive samples, although the percentages of positive samples were lower (**Figure 1**).

**Figure 2:**
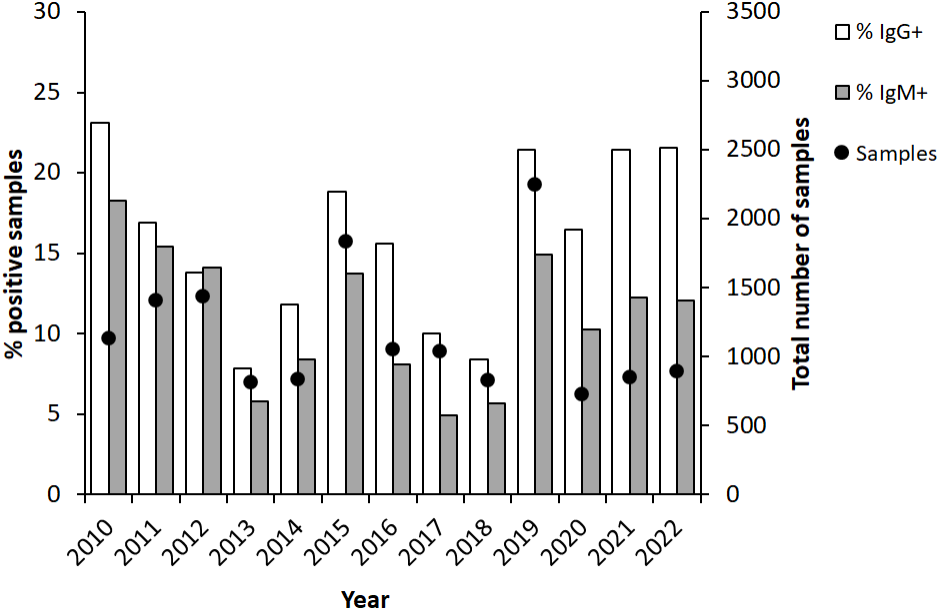
Annual proportion of IgG and IgM positive serum samples (bars) and numbers of serum samples analyzed each year (dot). The serum samples were collected from patients in Sweden with suspected tularemia during the period 2010 to 2022.

Analysis of the annual distribution showed variable numbers during the first half of the 12-year period, with the lowest in 2013, 812 samples, and the highest, 1,832, in 2015 (**Figure 2**). Peak numbers were recorded in 2019 when 2,250 samples were analyzed and then decreased to 700 - 800 between 2020 and 2022 (**Figure 2**). The percentage of IgG-positive samples was 23% in 2010 and decreased during the following years and was only 7.9% in 2013. Thereafter, the percentages of positive IgG samples gradually increased to 18.8% in 2015 and then decreased during a three-year period to reach 8.4% in 2018. In 2019, the year with peak number of samples, also a high percentage of the samples was positive, 21.4%. The same percentages were also observed in 2021 and 2022. A similar trend as described for IgG was observed for IgM-positive samples, although the absolute numbers and percentages of positive samples were lower (**Figure 2**).

In summary, high absolute numbers and peak percentages of positive samples appeared in 3 to 4-year cycles. Consistently, peak sampling had been conducted between July to November. The months with the highest percentages of positive samples were August-November.

### Age and gender effects on *F. tularensis*-specific antibody titers

**Figure 3:**
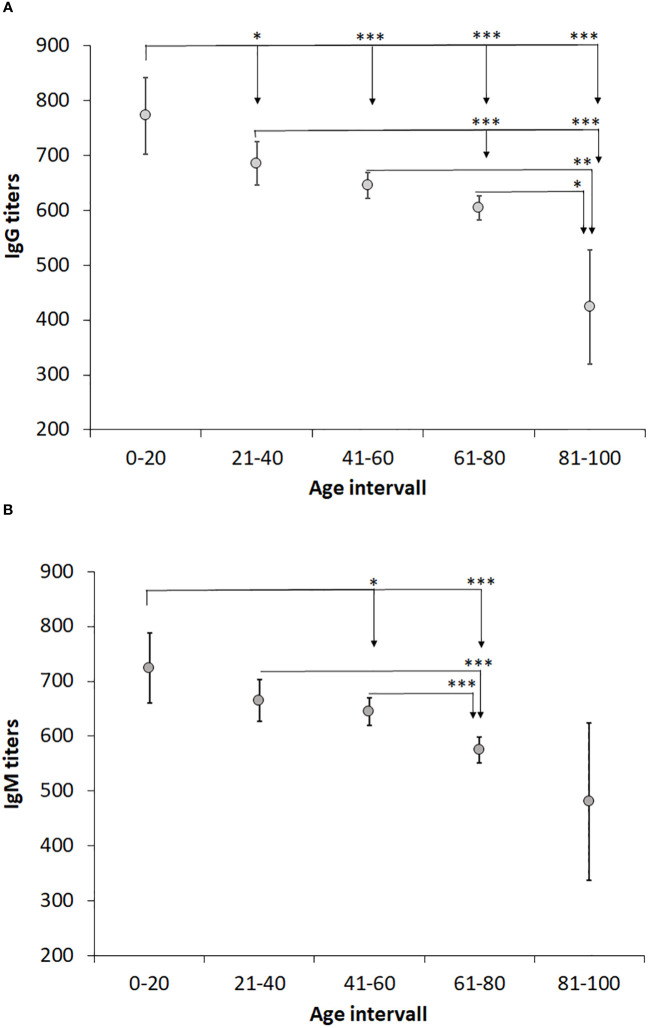
Analysis of the effect of age on IgG and IgM levels. Patients were divided into age groups with a 20-year interval and levels of IgG **(A)** and IgM **(B)** titers compared. One-way ANOVA with Bonferroni correction was used to assess differences in IgG and IgM levels between age groups. Significant differences are indicated by asterisks. *P < 0.05, **P < 0.01 and ***P < 0.001.

We investigated whether age influenced the IgG or IgM titers by comparing the average titers of each 20-year age interval (**Table 4**). Interestingly, the average IgG and IgM titers gradually decreased with increasing age (**Figures 3A, B**). In the age group 0 - 20 years, the IgG titer was significantly higher than in all other groups (*P* < 0.05; **Figure 3A**). The age group 21 - 40 years demonstrated higher average titer than the age groups 61-80 and 81 - 100 (*P* < 0.001; **Figure 3A**), but did not differ compared to age group 41 - 60. There was no difference between the groups 41 – 60 and 61 – 80, but both groups had higher average IgG titer than the oldest group (*P* < 0.05; **Figure 3A**). Similar age-related decreasing titers were observed for IgM and the average IgM titer of age group 61 -80 was lower than the younger groups (P < 0.05 – P < 0.001, **Figure 3B**). Age group 81 -100 showed great variation in IgM titers and, although the average titer was lower than any other group, it was not significantly lower than the other age groups (**Figure 3B**). Despite the gradually decreasing average titers with age, even in the highest age group, the titers were still much higher than the cutoff values of 200.

**Table 4:** Total and relative number of sera in each age and gender category.

Age interval	Total number	IgG-positive		IgM-positive	
		Female	Male	Female	Male
0 – 20	1,251 (8.3%)^1^	67 (0.4%)	74 (0.5%)	64 (0.4%)	72 (0.5%)
21 – 40	2,556 (17%)	175 (1.2%)	246 (1.6%)	130 (0.9%)	161 (1.1%)
41 – 60	4,826 (32%)	335 (2.2%)	590 (3.9%)	245 (1.6%)	418 (2.8%)
61 – 80	5,672 (38%)	259 (1.7%)	660 (4.4%)	194 (1.3%)	450 (3.0%)
81 – 100	680 (18%)	18 (0.1%)	40 (0.3%)	6 (0.04%)	15 (0.1%)
Total	14,985 (112^2^)	854 (58^3^)	1610	639 (46^3^)	1116

^1^Percentage of total number of samples.

^2^Number of samples missing age information.

^3^Number of samples missing the gender information.

The relative proportions of positive titers were analyzed per age group. With regard to IgG titers, a lower percentage was observed in the youngest and oldest age group, 11,3% and 8.5%, respectively, whereas the percentages of the other age groups were in the range of 16 – 19% (**Table 4**). With regard to IgM titers, again the oldest age group demonstrated the lowest percentage, 3.1%, whereas the other age groups showed percentages in the range of 11 – 14% (**Table 4**).

**Figure 4:**
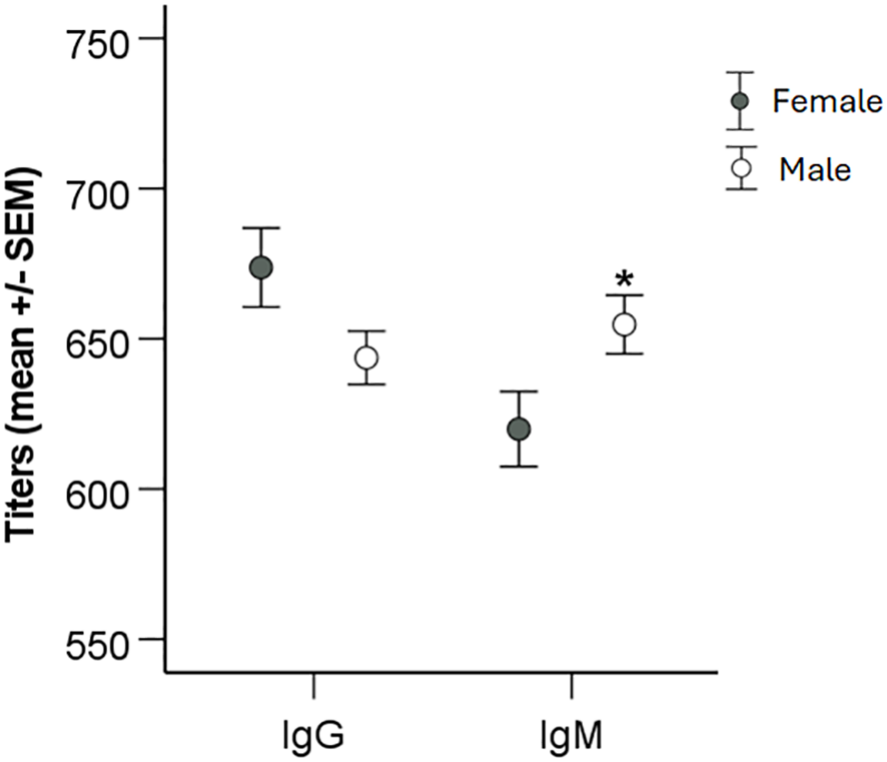
Analysis of the effect of gender on IgG and IgM levels. Patients were divided into groups based on gender and levels of IgG and IgM titers compared using two-sided Student`s *t*-test. Significant differences are indicated by asterisks. *P < 0.05.

**Figure 5:**
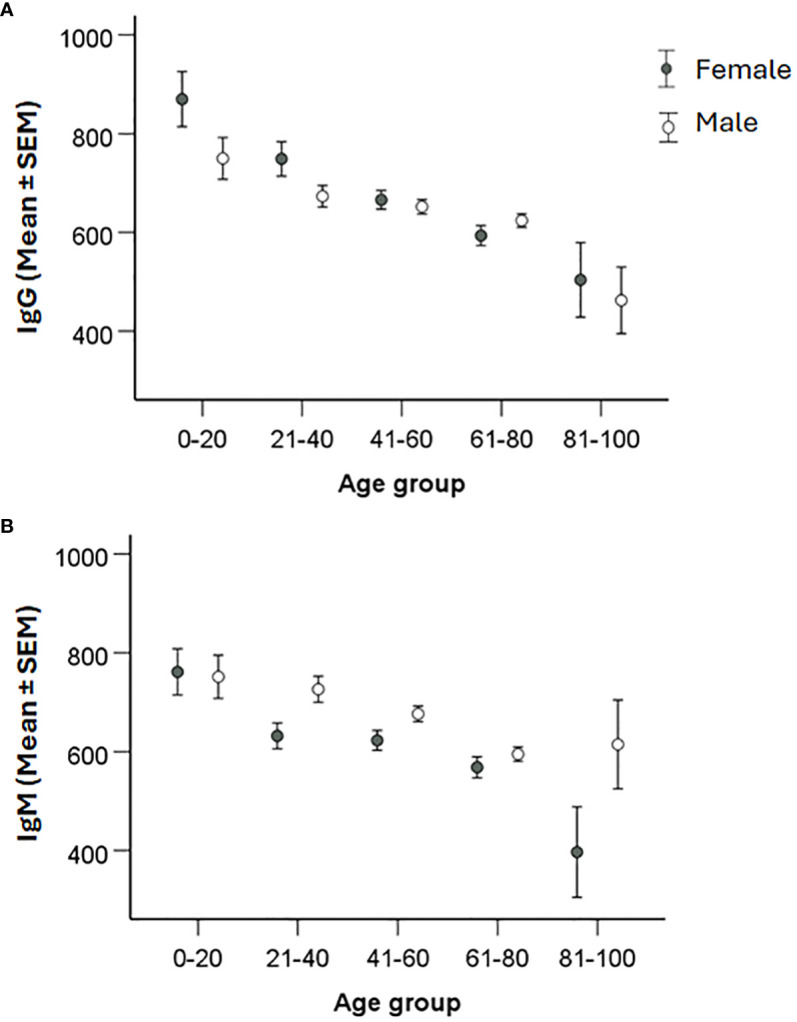
Analysis of the effect of gender and age on IgG and IgM levels. Patients were divided into 20-year age intervals and grouped according to gender. Levels of IgG **(A)** and IgM **(B)** between males and females in the different age groups were compared using two-sided Student`s *t*-test. Significant differences are indicated by asterisks. None of the gender differences was significant.

When titers were analyzed with regard to gender, it was observed that average IgM titers were lower in females than in males (*P* < 0.05), whereas there were no differences with regard to IgG titers (**Figure 4**). When average titers were grouped according to age intervals, there were no significant gender effects (*P* > 0.05; **Figure 5**).

Altogether, the data demonstrated that that gender influenced the IgM titers. IgG and IgM titers declined with age, however, regardless of age, average titers were still much higher than the cutoff values.

## Discussion

The utility of serology as the primary tool for diagnosis of tularemia is well established. Limitations with regard to other diagnostic techniques, such as PCR or cultivation, render serology the most practical diagnostic method, despite the slow kinetics with regard to antibody responses in general and especially with regard to tularemia (Maurin, 2020). An important aspect with regard to early detection of serological responses is the methodology. A number of comparative methodological studies have been performed during the last decade, including evaluations of current commercially available ELISA tests (Porsch-Ozcürümez et al., 2004; Chaignat et al., 2014; Yanes et al., 2018). One comprehensive study based on more than 100 sera observed that the ELISA tests detected antibodies earlier than indirect immunofluorescence assay (IFA) or microagglutination techniques (MAT), within 2 - 3 weeks after onset *vs*. 3 - 4 weeks for the latter methods (Porsch-Ozcürümez et al., 2004; Yanes et al., 2018). From a clinical perspective, this time difference is definitely valuable since it provides with considerably earlier diagnosis and is an important argument that a validated ELISA method should be one of the diagnostic methods utilized for the diagnosis of tularemia. Yanes et al. identified that the ELISA methods showed sensitivities close to 90% and specificities around 95% for both IgG and IgM antibodies. Repeated samples analyzed by ELISA demonstrated that 67% of samples were seropositive during the second week, and 100% during the third week (Yanes et al., 2018). Also, other studies have demonstrated that certain commercial ELISA tests have very good performance and are the preferred diagnostic methods (Maurin et al., 2011; Chaignat et al., 2014). Although several ELISA methods demonstrate good sensitivity and specificity, analysis of a high number of sera in countries with a very low incidence may still lead to a high false positive rate, since the specificities are around 95%, and under such circumstances using a complementary diagnostic serological method to validate positive findings is advisable.

Another aspect of clinical relevance is how long specific antibodies persist after treatment of infection. A recent study on tularemia patients demonstrated a very sustained persistence of both IgM and IgG antibodies, and significant decreases of IgG titers did not occur until 12 months after onset of disease and of IgM at 4 months after onset, but both IgG and IgM average titers still were above cutoff values at 12 months (Lindgren et al., 2022). Also, many other studies have observed long persistence of *F. tularensis*-specific antibodies. For example, a Finnish study evaluated the presence of antibodies in 23 tularemia patients up to 11 years past infection, and significant titers were found in all sera (Koskela and Salminen, 1985). Furthermore, a Japanese study and a Swedish study observed persistence of antibodies up to 20 and 25 years, respectively, after recovery from infection (Sato et al., 1990; Ericsson et al., 1994). Thus, this long persistence of *F. tularensis*-specific antibodies poses a diagnostic challenge and show that confirmation of a tularemia diagnosis either requires the demonstration of high antibody titers concomitantly with an acute disease presenting with symptoms typical of tularemia, or repeated serological investigation demonstrating significant decreases or increases of antibody titers in relation to a tularemia-like disease. Similar conclusions have been proposed by other authors (Syrjälä et al., 1986; Bevanger et al., 1988; Yanes et al., 2018).

The study demonstrated that a large majority of the serum samples analyzed did not exhibit significant IgG or IgM titers. When repeated sampling was performed, approximately a third of the originally negative samples showed seroconversion. Further analyses of these samples and the repeated samples that did not seroconvert, did not reveal any differences with regard to time to resampling, or the average age of the individuals of the two groups. The findings could be interpreted such that the clinicians’ diagnostic accuracy was low, however, in several areas of Sweden, the disease is intermittently relatively common and therefore, tularemia serology is routinely included when investigating, *e.g*., fever of unknown origin. Therefore, a lot of samples will be analyzed, even in the absence of any specific clinical suspicion, because tularemia will be an exclusion diagnosis and such samples can be part of the explanation for the high number of negative samples.

Our study demonstrated very significant variations in sampling during and between years. Since the laboratory serves as a national reference laboratory, the number of samples reflects the very substantial variation in reported Swedish tularemia cases. During the study period, there were fewer than 200 reported annual cases on five occasions, whereas the numbers of cases 2012, 2015, and 2019 were around 600, 900, and 1,000, respectively (Public Health Agency of Sweden, 2024). These three years were also those with the highest number of samples analyzed. Similarly, 3- or 4-year cycles of annual peak tularemia cases have also been reported from Finland and in Sweden during the 20^th^ century (Tärnvik et al., 1996; Rossow et al., 2015).

Interestingly, also the proportion of positive samples varied much between years. During years with peak number of samples, but also some years with intermediate number of samples, the proportion of IgG-positive samples was greater than, or close to 20%, whereas it was close to, or less than 10% during the years with the lowest number of samples. It is difficult to envision that these variations reflect changes in the work strategy of the clinicians, in particular since there was no time trend. Rather, it appears likely that it was an effect of the use of tularemia serology as a differential diagnostic measure in certain cases, *e.g*., investigations of unknown fever. Presumably, such investigations are rather constant over time and when the incidence of tularemia is low, then such analyses constitute a high proportion of all samples and the proportion of positive samples decreases. It should, however, be remarked that the experience of tularemia patients differs greatly between regions of Sweden. In endemic regions, each physician may encounter dozens or more patients in a given year, whereas in other regions of Sweden, some physicians may not have diagnosed any tularemia cases ever. Thus, the proportions of positive samples may also be dependent on the regional origin of the samples.

In this retrospective study, the number of samples during years also reflected the number of reported tularemia cases. A typical year, a majority of cases were reported during the period August-October. Also, monthly sampling showed similar variation and during August and September, there were > 3,000 monthly samples, more than 5 times as many monthly samples as during the period December - June. Higher numbers of samples were also recorded in July, October, and November, between 1,300 and 2,200. Thus, the sampling numbers closely correlated to the number of reported cases. The proportion of positive cases also varied much, being below or around 10% during the period February to July, around 15% in August, November, December, and January, and peaked in September and October, being > 20%. Again, the reasonable explanation is likely the same that may explain the pronounced variations in proportions of positive samples between years.

We had previously noted an age-related difference in IgG and IgM antibody titers in a small cohort of tularemia patients (Lindgren et al., 2022). Therefore, we analyzed the influence of age on the antibody titers and observed a marked decrease of titers in increasing age intervals, in particular significant for IgG titers, with the highest titers in the age group 0 - 20 years and lowest in the age group > 81 years. Of relevance, even in the highest age group, the average titers were considerably higher than the positive cutoff values. Although IgM titers were lower for females than males for the whole cohort, there were no gender differences noted in any of the age intervals. The significant inverse age-relation regarding antibody titers has been observed after various types of vaccinations, although findings are not consistent for all infections and vaccinations (Amanna et al., 2007; Frasca et al., 2011; Antia et al., 2018; Crooke et al., 2019). Presumably, such decreases reflect ageing of the immune system.

In conclusion, our study demonstrates a marked annual and seasonal variations in tularemia sampling occurring in Sweden and the most important explanation for this is the variation in reported tularemia cases nationally. Also, the proportion of positive samples increased during months and years with peak number of samples. A marked decrease in average antibody titers with increased age was observed.

## Data availability statement

The raw data supporting the conclusions of this article will be made available by the authors upon request. Requests to access these datasets should be directed to anders.sjostedt@umu.se.

## Ethics statement

Ethical approval for the study was received from the Swedish Ethical Review Authority, 2023-05165. The studies were conducted in accordance with the local legislation and institutional requirements. The samples had been collected as part of routine diagnostic analyses of patients suspected to have tularemia. Written informed consent for participation was not required from the participants or the participants’ legal guardians/next of kin in accordance with the national legislation and institutional requirements.

## Author contributions

HL: Conceptualization, Formal Analysis, Writing – original draft, Writing – review & editing. XL: Formal Analysis, Writing – original draft, Writing – review & editing. AS: Formal Analysis, Writing – original draft, Writing – review & editing, Conceptualization, Data curation, Funding acquisition, Resources.
